# A novel image database for social concepts reveals preference biases in autistic spectrum in adults and children

**DOI:** 10.3758/s13423-023-02443-7

**Published:** 2024-01-18

**Authors:** David Soto, Amaia Salazar, Patxi Elosegi, Antje Walter, Ning Mei, Ekaine Rodriguez, Valentina Petrollini, Agustín Vicente

**Affiliations:** 1 Basque Center on Cognition, Brain and Language, San Sebastian, Spain; 2 Ikerbasque, Basque Foundation for Science, Bilbao, Spain; 3 Department of Artistic Education, Complutense University of Madrid, Madrid, Spain; 4 University of the Basque Country- UPV/EHU, Leioa, Spain; 5 Shenzhen University, Nanshan District, Shenzhen, Guangdong Province China

**Keywords:** Social cognition, Abstract concepts, Preference biases, Metacognition, Autism

## Abstract

Human beings display the extraordinary ability of grasping and communicating abstract concepts. Yet, no standardized instruments exist to assess this ability. Developing these tools is paramount for understanding abstract representations such as social concepts, with ramifications in educational and clinical settings. Here, we developed an image database depicting abstract social concepts varying in social desirability. We first validated the image database in a sample of neurotypical participants. Then, we applied the database to test different hypotheses regarding how social concepts are represented across samples of adults and children with autism spectrum condition (ASC). Relative to the neurotypicals, we did not observe differences related to ASC in identification performance of the social desirability of the concepts, nor differences in metacognitive ability. However, we observed a preference bias away from prosocial concepts that was linked to individual autistic traits in the neurotypicals, and higher in ASC relative to the neurotypicals both in adults and children. These results indicate that abstract representations such as social concepts are dependent on individual neurodevelopmental traits. The image database thus provides a standardized assessment tool for investigating the representation of abstract social concepts in the fields of psycholinguistics, neuropsychology, neuropsychiatry, and cognitive neuroscience, across different cultures and languages.

**Supplementary Information:**

The online version contains supplementary material available at 10.3758/s13423-023-02443-7.

## Introduction

The past two decades have witnessed a flourishing interdisciplinary interest in the study of the brain representations of meaning, bridging across the fields of psychology, linguistics, cognitive neuroscience, and artificial intelligence. This research has mainly focused on concrete concepts. Human beings, however, display the extraordinary ability of representing and communicating abstract knowledge. Only recently has there been an attempt to study the representations of abstract concepts such as emotions, personality traits (Alcalá-López & Soto, 2020), social concepts (Conca et al., 2021; Pexman et al., 2023), or aesthetic notions such as “beauty” or “justice” (Borghi et al., 2018). Originally, semantic representations were thought to be amodal and abstract concepts were considered purely linguistic forms (Barsalou, 2020). Unlike concrete concepts, abstract concepts are not bound to any physical perceptual referent (Borghi et al., 2017). According to the grounded cognition framework, abstract concepts involve complex, integrated representations of events or situations that can only rely on perceptual and action systems to a certain degree (Barsalou, 2020; Borghi et al., 2017; Wilson-Mendenhall et al., 2011).

Currently, there are no standardized tools for studying abstract social concepts. This lack of tools can lead to mixed results due to different criteria for the selection and depiction of abstract concepts (Villani et al., 2019). Images can serve as a means to depict abstract concepts as a situated experience. More concretely, by integrating situational components, image-based conceptualizations can provide richer representations that afford the re-enactment of similar situations and scripts, thereby providing a unique opportunity to study the mechanisms involved in the grounding of abstract representations. Here, we developed a novel image database to assess the representation of social concepts. Past psychological research emphasized how human judgements relative to others’ behavior are influenced by their social desirability (Anderson, 1968; Fisher et al., 1985; Norman, 1967). Accordingly, the newly devised image database included abstract concepts differing in social desirability, half of which were highly likable and prosocial (e.g., ’grateful’, ’polite’, ’witty’) and the other half were socially undesirable (e.g., ’jealous’, ’rude’, ’messy’), following a prior norming study (Anderson, 1968). Developing this type of tool is paramount for understanding how social concepts are represented, with ramifications in educational and clinical settings. For instance, difficulties in social interaction in conditions such as autism spectrum condition (henceforth ASC) may partially relate to the inability to track and simulate abstract concepts related to others’ actions, emotions, and viewpoints (Matheson & Barsalou, 2018)

.

Here, we present the results of two experime nts applying the database to test the hthesis that the representation of social concepts depends on individual neurodevelopmental traits linked to ASC. ASC is characterized by difficulties in evaluating social cues and understanding the emotions, intentions, and mental states of others (Dapretto et al., 2006; Król & Król, 2020), even in individuals who are verbally and intellectually within typical parameters (Baron-Cohen et al., 2001; Shulman et al., 2012). Impaired social attention mechanisms in ASC (e.g., difficulties in joint attention) may lead to reduced interest in social interaction (Baron-Cohen, 1997; Chevallier et al., 2012). Notwithstanding, the existence of difficulties in abstract conceptual processing in ASC is under debate (Borghi et al., 2021) with several studies showing that ASC individuals can exhibit relevant skills for social cognition, such as mental state recognition, or social and moral reasoning (Blair, 1996; Carpenter et al., 2001; Grant et al., 2005; Russell & Hill, 2001; Shulman et al., 2012). Hence, despite difficulties in social attention (Dawson et al., 2004), abstract social conceptualizations in ASC may be preserved. Here, we developed an odd-one-out search task to assess the perceptual identification of social desirability in ASC.

Finally, we developed a choice preference task with items competing in social desirability to assess whether autistic traits influence subjective biases over and above the discrimination of the social desirability. Previous research has shown preference biases in ASC for nonsocial (i.e., videos of geometrical figures) over social stimuli involving people (Crawford et al., 2016; Gale et al., 2019; Król & Król, 2020). Here, we tested preference biases within the social domain, namely, for concepts differing in social desirability. ASC samples of both adults and children were included because we were interested in understanding how the concepts related to social behavior across development. The development of the image database allowed devising tasks, such as the preference task, which could be administered to young children without requiring verbal elaboration or complex interaction. Similar tasks assessing early social and moral capacities have been administered to neurotypical infants (6–10 months of age) as well as to toddlers between 19 and 23 months of age (Hamlin & Wynn, 2011; Hamlin et al., 2007). We were particularly interested in determining whether the preference bias differs across development, namely, whether the expected pattern of prosocial choices in adulthood was already present in children and whether the preference bias reflects a stable trait in autism across development.

## Methods

### Participants

All adult participants were recruited via the Internet platform Prolific and received a monetary remuneration of £7 per hour. Prolific offers several filter criteria for participant screening. The following filters were used: a) English as a first language, b) no literacy difficulties, or c) mild cognitive impairments (MCI) or dementia, and d) normal or corrected-to-normal vision. To reinforce the probability of obtaining quality submissions, we additionally indicated a participant approval rate of 100% and a minimum of 50 prior submissions. We made no specifications regarding educational background or socio-economic status, as prior studies have shown that neither of these factors is significantly related to the presence or absence of autistic traits in the general population (Baron-Cohen et al., 2001). Also, participants in the neurotypical group should not have been officially diagnosed with ASC. The children were recruited at a local school (see further below). Ethical approval was issued by the Local Research Ethics Committee.

We included a bigger size in the neurotypical sample in order to validate the database and also because we aimed to assess inter-individual variation in autistic traits and task performance. A power analysis using G*power (Faul et al., 2007) was conducted to determine the minimum sample size required to achieve a correlation of 0.2 between individual ASC trait and performance in the odd-one-out and preference tasks with $\alpha$ = .01 and an expected power (1 - $\beta$) = .80. Results of this power analysis showed that 247 participants would be needed in this case. We topped this number up a little to mitigate any potential data loss that could arise in online studies.

Regarding the comparison of preference biases between the ASC and the neurotypicals, a power analysis using G*power (Faul et al., 2007) was run. Gale and colleagues (2019) reported an effect size of d = 0.871 for the comparison of the social vs. non-social choice bias in children with ASC relative to controls. Accordingly, a minimal sample size of 32 participants was necessary to achieve a similar effect in an independent-samples *t* test comparing ASC and control samples with $\alpha$ = .01 and an expected power (1 - $\beta$) = .80 was. However, since we also used an adult sample for this comparison, we elected to increase the sample to 50 participants to be able to detect even a smaller effect. The sample size of the ASC children sample was constrained by the number of families with ASC children that we were able to approach.

Regarding the male-female ratio in the Prolific adult ASC sample, we noted that 33 subjects were females and 16 were males (gender data from one participant was missing). We acknowledge that the sex ratio of our ASC adult sample is unusual, given that the male-to-female ratio for autism is typically estimated to be 4:1 (CDC, 2020). Yet, some epidemiological studies suggest that the ratio might actually be closer to 2:1 (Kim et al., 2011; Mattila et al., 2011). Recent evidence also shows that the male-to-female ratio decreases with age (Posserud et al., 2021), possibly as an effect of later diagnosis in females (Bargiela et al., 2016). The higher-than-average percentage of female respondents in our adult sample may thus reflect a general trend in recent online studies on autistic adults, which tend to attract more female participants overall (Hull et al., 2017; Livingston et al., 2019). Given that females are still overwhelmingly underrepresented in autism research (D’Mello et al., 2022), our high percentage of female participants proves valuable to collect data on a historically understudied population.

**Neurotypical subjects** We were able to retrieve the data of 255 participants from the neurotypical group. Twenty-five subjects could not complete the final test involving the Autism-Spectrum Quotient (AQ) questionnaire due to a technical issue that sometimes occurred when redirecting to the URL of this task component. Two further subjects were excluded due to failed attention checks and a completion time longer than two standard deviations from the average. Hence, a total of 228 subjects completed all four tasks and these data were used to examine inter-individual differences in autistic traits and behavioral scores in the odd-one-out and preference tasks (see below for descriptions of these tasks). However, those participants that could not complete the AQ questionnaire were still included in the analyses of the semantic decision task to validate the image database. The neurotypical subjects included in the final data set had a mean age of 41.53 (SD = 12.79, range 19–79), 139 subjects were females. Of the total sample, 89% was right-handed.

**ASC adult sample** We recruited 50 adult participants from Prolific that indicated having received a diagnosis of ASC as an adult or as a child. The mean age of the ASC group was 33 (SD = 9.48, range 19–61). One participant could not complete the Autism-Spectrum Quotient (AQ) questionnaire for the reasons given above. Thirty-three subjects were females, and 16 were males (gender data from one participant was missing). Ten percent of the participants were left-handed.

**Children: Neurotypicals and ASC samples**

Typically, developing children (*N* = 29) were recruited at a local school, with the ages of 4, 5, and 6. Parents of these children received an invitation letter from the school management team, attaching the informed consent form. The experimental task was administered in facilities provided by the school. The sample of neurotypical children was formed by three age groups: 4-, 5-, and 6-year-olds, with nine children in the younger group and ten in each of the other groups.

The sample of ASC children (*N* = 16) comprised ages from 4 to 9 (three children of 4, two of 5, nine of 6, one of 8, and one of 9). None of them exhibited a concomitant intellectual disability, although there was a great degree of variability in this respect (LEITER-3 scores going from 73 to 125). ASC children were matched as closely as possible with neurotypical children by verbal mental age (VMA) measured with the PPVT-3. This ranged from 3;2 (3 years, 2 months), to 7;6 (7 years, 6 months). All of them had either an autism diagnosis or were tested by a clinician in one of our labs with the ADOS-2 test. ASC children (*N* = 16) were recruited by one of our labs, where we work with several families and we routinely administer children several tests, including the ADOS-2 (Lord et al., 2002). We contacted 17 families. Inclusion criteria were: having a verbal mental age (VMA) between 3 and 8 years, and not having a concomitant intellectual disability (the lowest NVIQ was 73, and it was the only borderline one). Only one child was excluded due to lack of attention and/or restlessness that precluded him from completing the task. Children were tested in the lab by an occupational therapist they were already familiar with, being the person who also administers the ADOS test. Families were invited to stay in the room while watching from a distance without intervening.

### Image database

The newly developed database comprised a total of 60 images depicting a social concept associated with a personality trait. The concepts were selected from a previous norming study performed with abstract adjectives (Anderson, 1968). We selected 60 trait adjectives, 30 exhibiting a high social desirability (normative ratings $> 350$ in Anderson’s study (Anderson, 1968)) and 30 exhibiting a low social desirability (ratings $< 350$). The concepts were visualized in 60 illustrations using situated conceptualizations. Each image represented a concrete experience associated with the specific concept. The illustrations were created on the basis of a series of common characteristics. All images were elaborated as homogenous black-and-white drawings to avoid diverting the viewer’s focus on the illustration, as it could happen in case of adding intense primary or secondary colors of the chromatic range. Simple gray-scaled shadows were added in an aquarelle-like technique to mark the ground and indicate the specific physical position of the protagonist’s bodies in the situation. The visual representation of an abstract concept is made through figurative identification based on embodied non-verbal language of body posture. A concrete action was represented through static images of human figures. Human figures have been depicted as gender-neutral without eyes, and the lowest possible degree of detail. Relevant social information was indicated through bodily postures and gestures and, where necessary, facial expressions were indicated through the mouth as a transmitter of linguistic information and emotions during social interaction. A red arrow indicated the protagonist whose behavior referred to the target concept. The abstract concepts are represented through actions at a specific moment taking place in a single scene without sequencing or staged series (e.g., comic). The images are based on perceptual-motor aspects related to the experience of the body within the drawn space (Merleau-Ponty, 1945). These can be found in the Supplementary Figures and also at the OSF website linked to the project (https://osf.io/6myaf/).

### Computer vision model representations of the images

Images were quantified by fine-tuning a computer vision model (Yosinski et al., 2014) pretrained with the ImageNet dataset (Deng et al., 2009). The model backbone chosen was ResNext101 (Xie et al., 2017). This choice was based on a Kaggle^1^ blog post^2^ (Rwightman, 2019) indicating that ResNext models outperformed all other models. The pretrained ResNext101 model was downloaded from PyTorch V1.8.0 and Torchvision V0.9.0 (Paszke et al., 2017, 2019). The convolutional layers of the ResNext101 model were frozen and their weights were not updated during the fine-tuning phase. An adaptive pooling (McFee et al., 2018) operation was applied to the last convolutional layer. A new fully connected layer received the weighted sum of these outputs (i.e., the hidden layer). The size of the hidden layer was 300 units and its output was passed onto a nonlinear activation function (Specht, 1990), namely, the self-normalized rectification function (SELU, Klambauer et al., 2017). A new fully connected layer (i.e., the classification layer) took the SELU outputs. The size of the classification layer was 1000 for the 1000 categories in the ImageNet-sketch dataset. During this fine-tuning, a batch size of 16 was used, the loss function was binary cross entropy, and the optimizer was stochastic gradient descent with a learning rate of $1^{-4}$. The ImageNet-sketch dataset was randomly split into training and validation sets. If the validation loss did not improve for ten consecutive epochs, the training was terminated.

Then, the 60 images were fed to the trained model, and the hidden layer representation (before the classification layer) and also the first layer representations were recorded for future analyses. In order to reduce the dimensionality of the first layer representations of the images, the representations were flattened from the original three dimensions (220 $\times$ 220 $\times$ 128 $\rightarrow$ 6,195,200) and a principal component analysis (PCA) implemented in Scikit-learn V 0.24 (Abraham et al., 2014; Pedregosa et al., 2011) was applied to the flattened representations to reduce the dimensionality to 300. To further assess the computer vision representations of the images, we conducted a decoding analysis and a simple representational similarity analysis (RSA, Diedrichsen and Kriegeskorte (2017); Kriegeskorte et al. (2008)) to the representations.

The decoding analysis used the computer vision model representations to predict the social desirability (low vs. high) of each image using a leave-two-words-out cross-validation procedure. In each cross-validation fold, one low and one high social desirability were left out as the test set, and the rest were used to train a linear support vector machine (SVM) classifier. The predictions obtained in the test set were compared to the true labels by means of a receiver operating characteristic curve (ROC AUC). The procedure returned 900 folds of cross-validation ROC AUC scores (the experimental scores). Then, we conducted a permutation test (Ojala & Garriga, 2010) using the same cross-validation procedure, except that the correspondence between the representations and the labels was shuffled in the training set (not modified in the test set). The average of the cross-validation ROC AUC scores was used as an empirical chance level estimate. The permutation test was repeated 1000 times to estimate a distribution of the empirical chance level. The statistical significance was measured by the probability of the empirical chance level being greater or equal to the average of the experimental scores.

Besides the decoding analysis, a simple RSA was used to visualize the relationship among the different representations of the 60 concepts. A representational dissimilarity matrix (RDM) was computed based on each pair of the representations of the 60 images. The RDM was measured by 1 - Pearson correlation.

## Apparatus

The tasks were programmed in OpenSesame 3.3.12 Lentiform Loewenfeld (Mathôt et al., 2012) and the resulting OSWeb scripts were imported to JATOS 3.7 (Lange et al., 2015) to perform the study online. A web link was provided to the study participants on the platform Prolific.

## Tasks and experimental procedure

Participants performed three experimental tasks plus the AQ questionnaire in the following order:

### The semantic-decision-task

Participants were shown a single image centered in the middle of the screen. Each image contained a red arrow pointing to the protagonist whose behavior related to the target concept. Two words appeared, one to the left and one to the right of the image. One word referred to the intended target-concept. The distractor word referred to another personality trait word from the database of opposite social desirability (i.e., if the target-concept was socially desirable, the distractor word referred to a socially undesirable concept and vice versa). Participants had to decide which of the two provided concepts was depicted in the presented image. If the target-concept appeared to the left, subjects were to press the ‘z’ key, if it appeared to the right, the ‘m’ key. Following this, participants were asked to provide different ratings using a ten-point rating scale. To answer, participants were instructed to move the slider cursor either to the left by pressing the ‘z’ key or to the right by pressing the ‘m’ key. The response had to be validated by pressing the space key and only then did the screen changed to the next question. Rating questions were as follows: “How well does the image illustrate the meaning of the word you have chosen?” “Please indicate the degree of emotionality expressed by the image.” “Is this concept socially acceptable?” The semantic-decision-task consisted of 60 trials, one per image, in a randomized order.

### The odd-one-out-task

Three images were simultaneously displayed on the screen. Two images were selected from the same social desirability status, whereas the third image was the odd-one-out. If the two matching images depicted a socially desirable concept, the third image depicted a socially undesirable concept and vice versa. The protagonist of each image was indicated by a red arrow. Importantly, participants were only told that two images were more related, whereas a third one was “more like a singleton” but without making the social desirability factor explicit. The task was to indicate the location of the odd-one-out (i.e., left, top-middle, right) by pressing the ‘z’, ‘v’ and ‘m’ keys. Following this, they had to rate their confidence in their decision using a four-point scale ranging from ‘guess’ to ‘sure’. The odd-one-out-task consisted of 60 single trials in randomized order with each of the 60 images being the odd-one-out across the trials. Figure 5 illustrates an example trial of the odd-one-out-task.

**Fig. 1:**
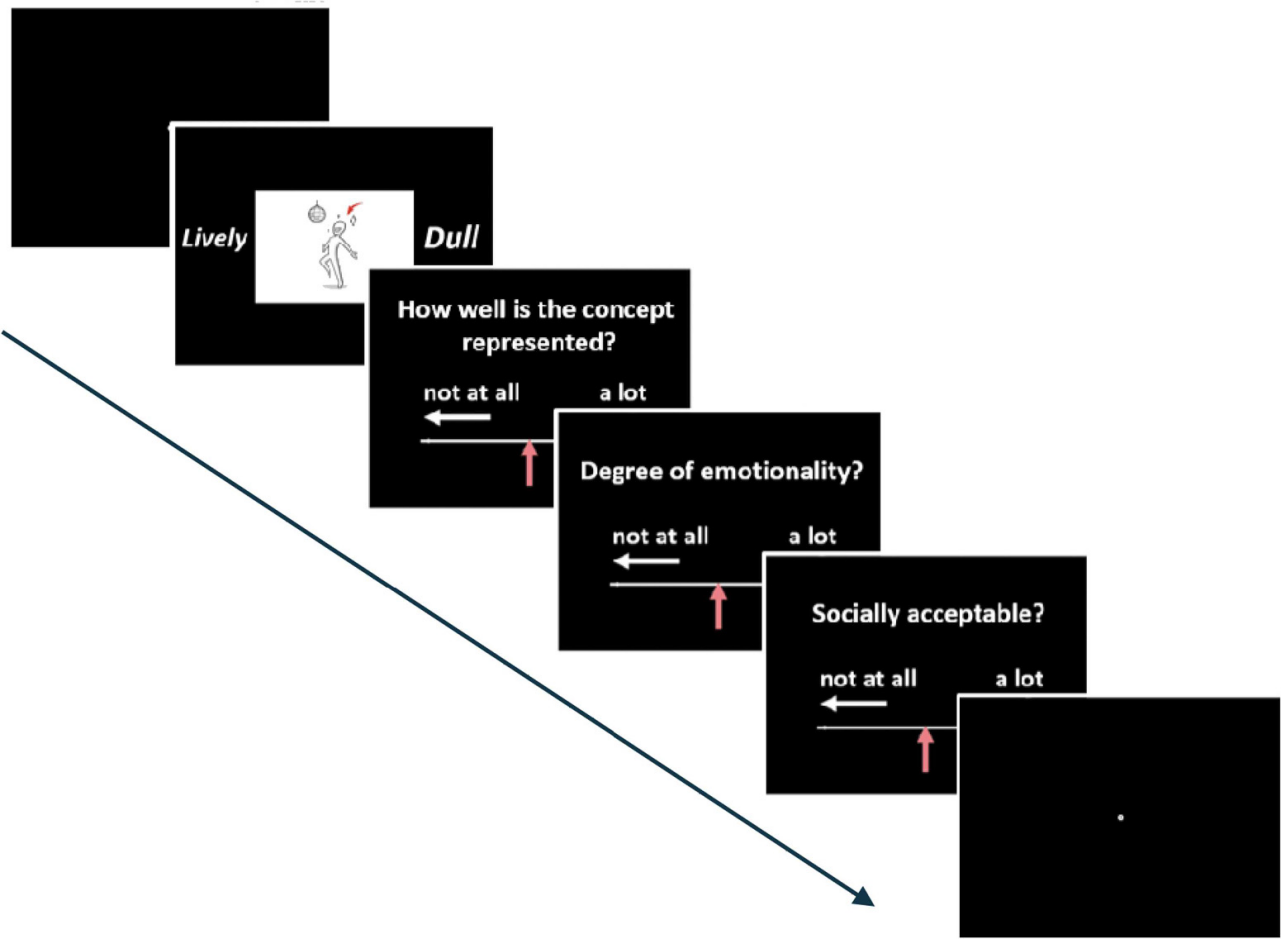
Illustration of the experimental procedure in the semantic decision task. Participants were shown a single image in the center, flanked by two words. They had to decide which of the two words was depicted in the presented image. Following this, participants rated how well the image illustrated the meaning of the selected word, the degree of emotionality expressed by the image and the social desirability of the concept. The semantic-decision task consisted of 60 trials, one per image, in a randomized order

### The preference task

Two images were simultaneously presented on the screen, one to the left, and one to the right. One image depicted a socially desirable concept and the other depicted a socially undesirable concept. Participants had to indicate whether they subjectively preferred the protagonist in the image to the left (z-key) or to the right (m-key). The protagonist of each image was indicated by a red arrow. Following this, participants made a second more fine-grained rating using a ten-point scale ranging from “not at all” to “a lot”. The preference task comprised 30 trials in randomized order.

### The Autism-Spectrum Quotient

Finally, adult participants completed the Autism-Spectrum Quotient. The AQ contains a total of 50 statements with four response options. Participants were presented with the respective statement on the upper part of the screen and the response options below. By pressing either the key number 1, 2, 3, or 4, subjects indicated whether they 1 = definitely agreed, 2 = slightly agreed, 3 = slightly disagreed or 4 = definitely disagreed with the presented statement.

We computed the individual AQ scores in conformity with the pre-established norms. Namely, for the queries 2, 4, 5, 6, 7, 9, 12, 13, 16, 18, 19, 20, 21, 22, 23, 26, 33, 35, 39, 41, 42, 43, 45, 46 the answers “slightly agree” and “definitely agree” score 1 point, whereas the responses “definitely disagree” or “slightly disagree” to queries 1, 3, 8, 10, 11, 14, 15, 17, 24, 25, 27, 28, 29, 30, 31, 32, 34, 36, 37, 38, 40, 44, 47, 48, 49, 50 score 1 point. The total of points obtained determines the individual AQ score.

### Statistical analysis

Statistical analyses were performed using custom-made scripts written in Python 3.9.7 and JASP 0.16.3. The normality assumption of the data across the sample groups was checked by using the Shapiro–Wilk test of normality, and when a deviation from normality was detected, we followed up with a non-parametric version of the relevant statistical test (i.e., the Mann–Whitney *U* test for independent sample comparisons). We used a two-tailed alpha level of .05 for all statistical tests.

## Results

### Image database validation

First, we determined whether the image database (see Supplementary Figures 1 and 2) accurately depict the target concept, and whether the images reflected the social desirability established by Anderson’s normative study (Anderson, 1968). Two hundred twenty-eight participants performed a semantic decision task (see Fig. 1)in which they had to decide which of the two flanking word concepts was depicted by the image and, subsequently, rate the image’s social desirability and then provide ratings of semantic relatedness, emotion, and social desirability. Emotional ratings were not analyzed in the context of the present study because we did not have an priori hypothesis regarding how the emotionality ratings may vary across ASC traits in the neurotypical sample. However, the data has been made open-access and researchers may be able to use this information in future studies.

**Fig. 2:**
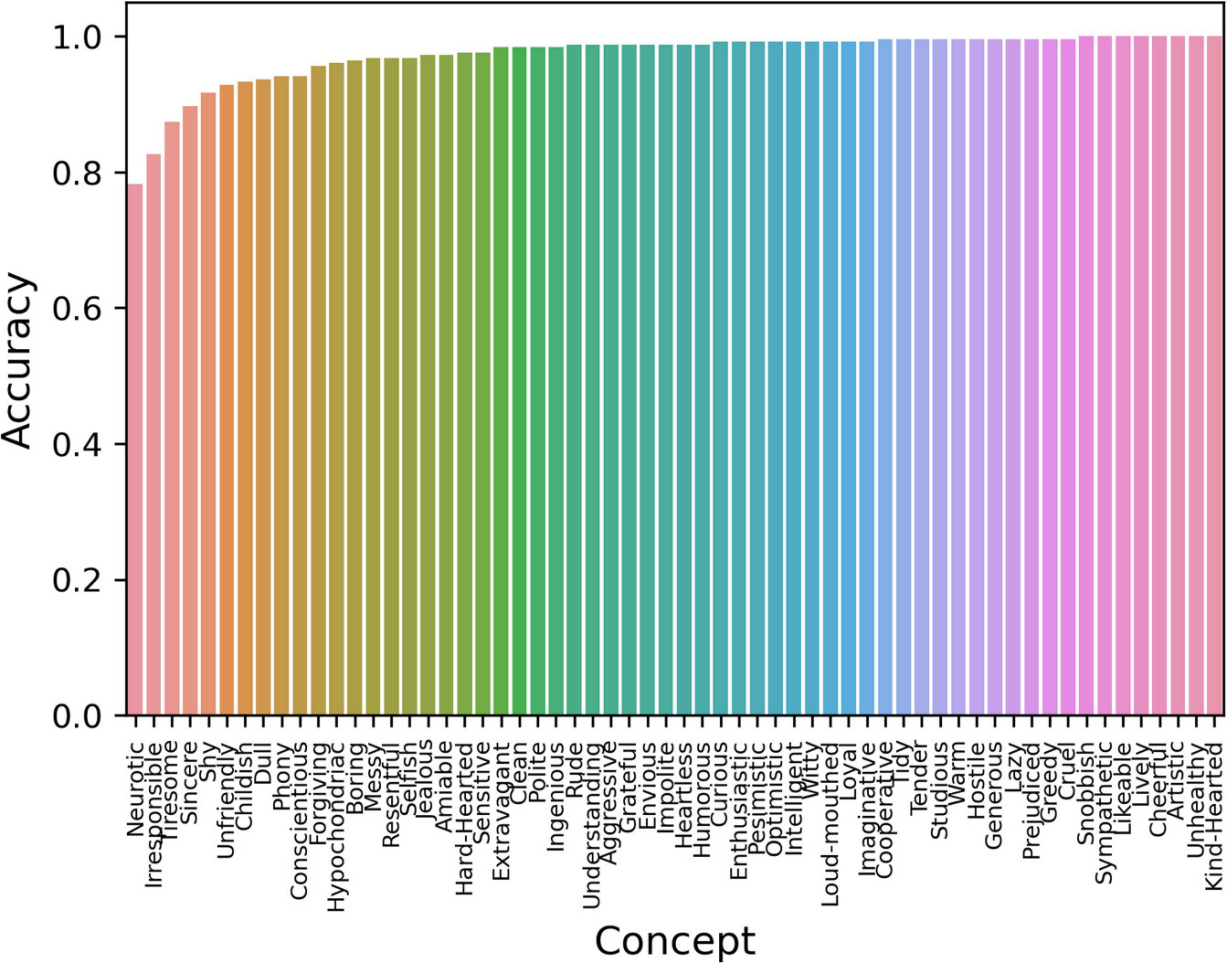
Probability of selecting the target concept across participants

**Fig. 3:**
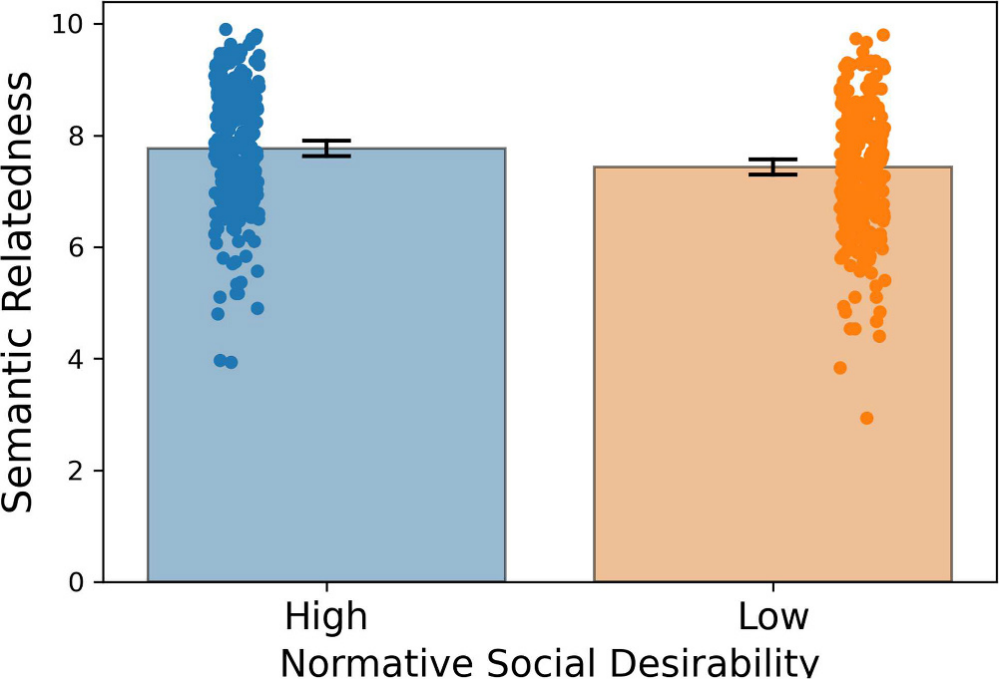
Mean semantic relatedness ratings as a function of the normative social desirability of the items. The *bars* represent the 95% confidence intervals around the mean

Figure 2 illustrates the mean probability of selecting the target concept across participants, for each of the concepts of the database. With the exception of the adjective “neurotic” (probability of 0.78), the mean probability that the target-concept was very high. We further observed that the participants’ semantic relatedness ratings between the selected word concept and the image were consistently high across participants (Fig. 3; Supplementary Figure 3 depicts the average semantic relatedness rating for each of the concepts across participants). The paired-samples *t* test revealed that semantic relatedness ratings were slightly but significantly higher for socially desirable relative to social undesirable concepts (*t*(227) = 8.396, $p < 0.0001$, Cohen’s *d* = 0.528).

We then examined whether the subjective ratings reflected the normative social desirability following Anderson’s norming study (Anderson, 1968) by comparing the mean social desirability ratings between the high and low normative desirability items. A two-sided *t* test demonstrated significantly higher desirability ratings for the socially desirable images (t(252) = 55.52; $p < 0.001$, Cohen’s *d* = 3.49).

### Computer vision model representations of the images

We used computer vision models to extract representations of the 60 images. The model backbone was based on the convolutional layers of Resnext101 Xie et al. (2017), which was pretrained using the ImageNet dataset (Methods). The model representations can be found in the OSF website linked to the project. We performed a decoding analysis on the hidden layer and also on the first layer representations of the 60 images. A linear support vector machine was used to classify the social desirability of the images (i.e., high vs. low). The results showed that social desirability appeared to be decoded from the hidden layer (ROC AUC $=$ 0.59 ± 0.12, $\mu \pm \sigma$, *p* = 0.0238, Supplementary Figure 4) and also from the first layer representations (ROC AUC $=$ 0.59 ± 0.12, $\mu \pm \sigma$, *p* = 0.0244, Supplementary Figure 5). However, we note that although the statistical results were reliable, the sample size of the cross-validation was only 60. Therefore, statistical inference might be inflated by the small sample size and the dependence on outliers in the cross-validation. The large standard deviation in the ROC AUC scores are in keeping with this possibility and hence the possibility that the social class of each concept can be predicted by specific visual features in the images should be taken with caution. We also computed the representational dis-similarity matrix for the images and there were no visible clusters that differentiated low and high desirability (Supplementary Figures 6 and 7).

**Fig. 4:**
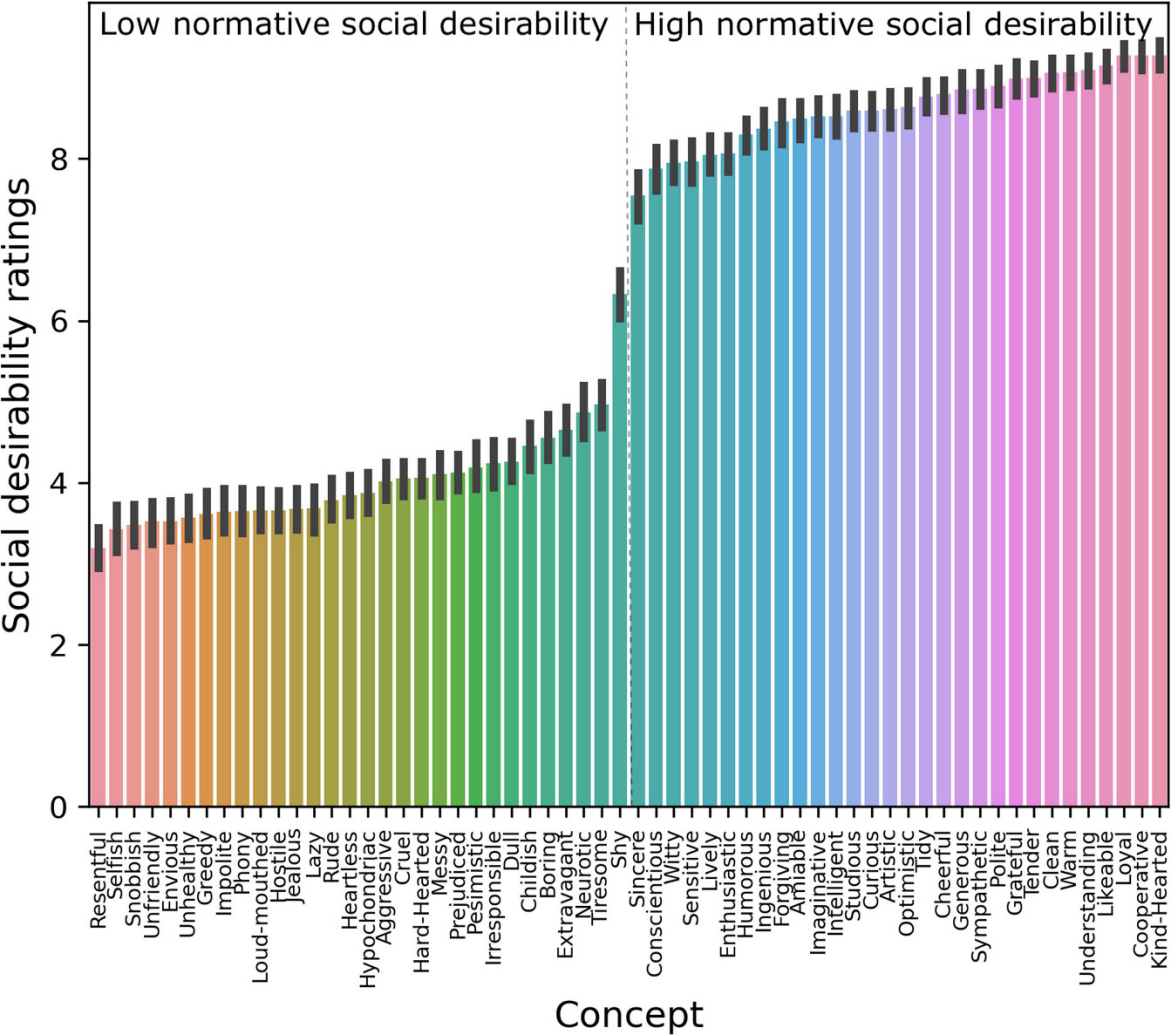
Mean social desirability ratings for each concept across subjects. The concepts have been split by the normative social desirability following a prior norming study (Anderson, 1968). The *bars* represent the 95% confidence intervals around the mean

### Applying the image database to assess discrimination of social desirability in ASC

**Fig. 5:**
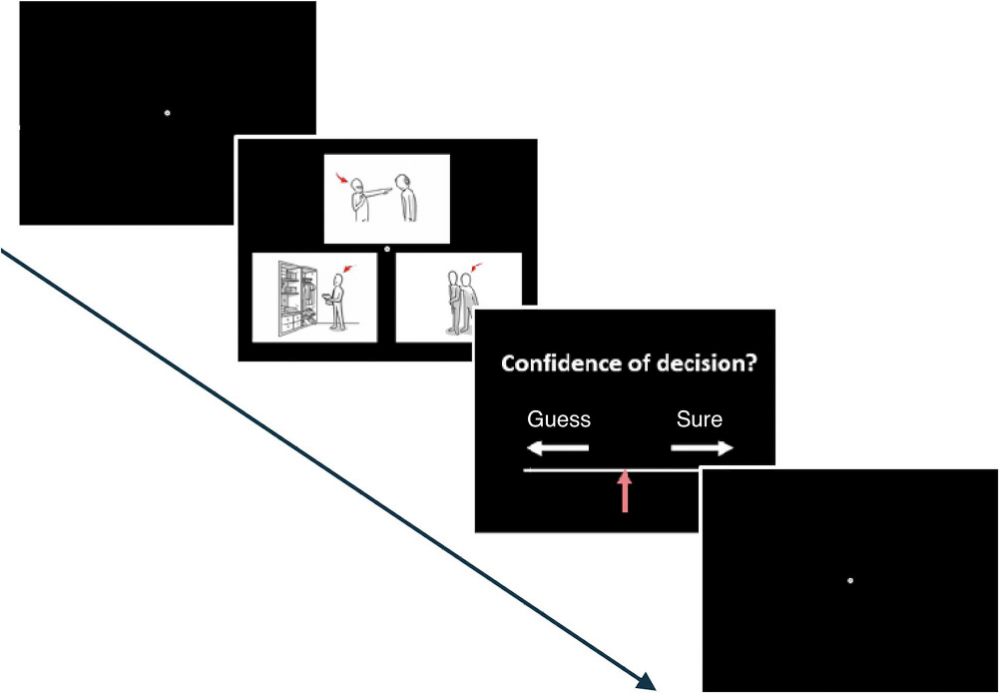
Example trial of the odd-one-out task. Three images were simultaneously displayed on the screen. Two images were selected from the same social desirability status, either high or low, whereas the third image was the odd-one-out. The task was to indicate the location of the odd-one-out by pressing one of three response keys

**Fig. 6:**
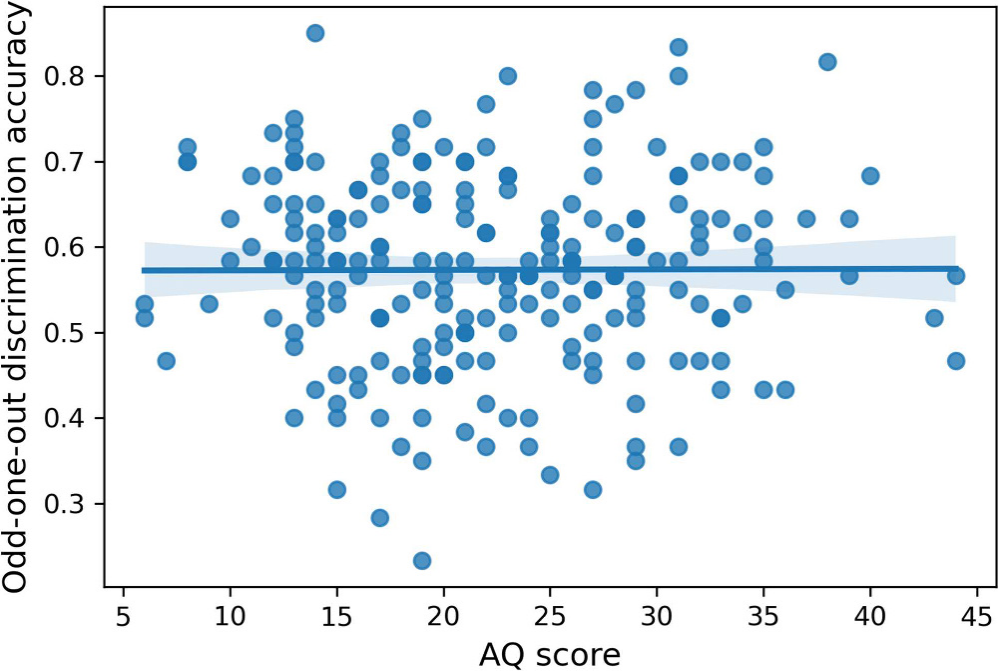
Scatterplot showing the absence of correlation between the AQ scores and odd-one-out-task performance of the neurotypical subjects

Participants performed a search task in which they had to identify which of three presented images was the odd-one-out in terms of social desirability level (see Figs. 4 and 5). Figure 6 shows that neurotypical participants performed consistently above the 0.33 chance level. Spearman’s correlation between the Autism-Spectrum Quotient (henceforth AQ) score of the neurotypical group and the individual accuracy revealed no association between the two, *r* = -0.22, $p = 0.74$. Figure 6 depicts these results.

**Fig. 7:**
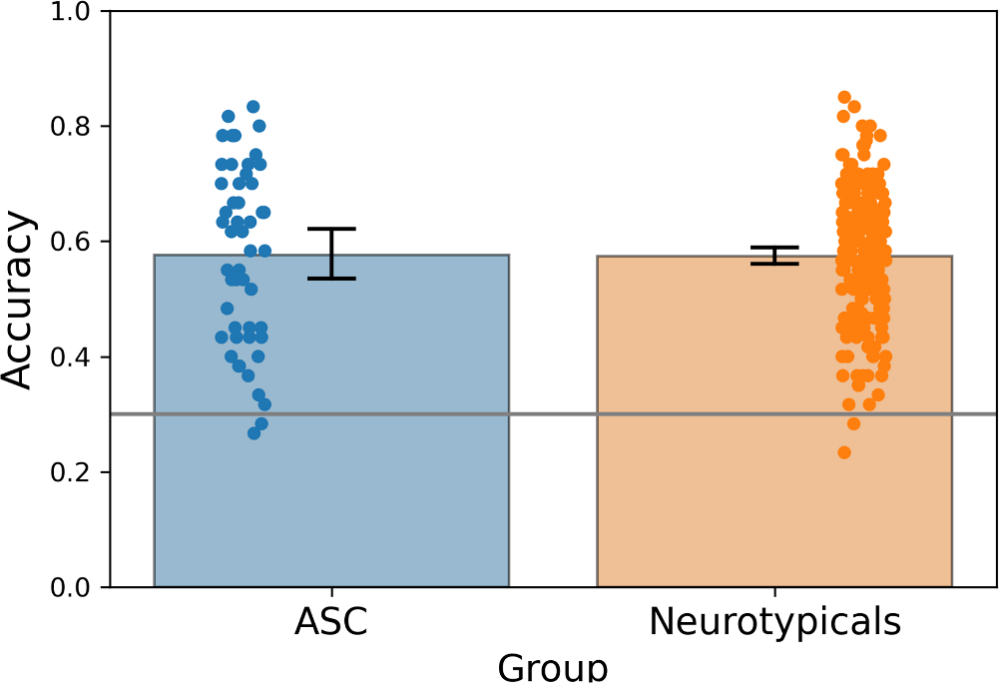
Mean discrimination performance in the odd-one-out-task across the ASC and neurotypical groups. The *bars* represent the 95% confidence intervals around the mean

We confirmed that the ASC sample had a higher AQ score compared to the neurotypicals (t(275) = 11.327, $p < 0.001$, Cohen’s *d* = 1.799, AQ data from one ASC participant was missing). ASC participants performed consistently above chance level in the odd-one-out task, with a level of performance that matched the neurotypical sample (*t*(276) = 0.151, $p = 0.88$; Mann–Whitney test, *U* = 5904, $p = 0.692$). Figure 7 illustrates these results.

**Fig. 8:**
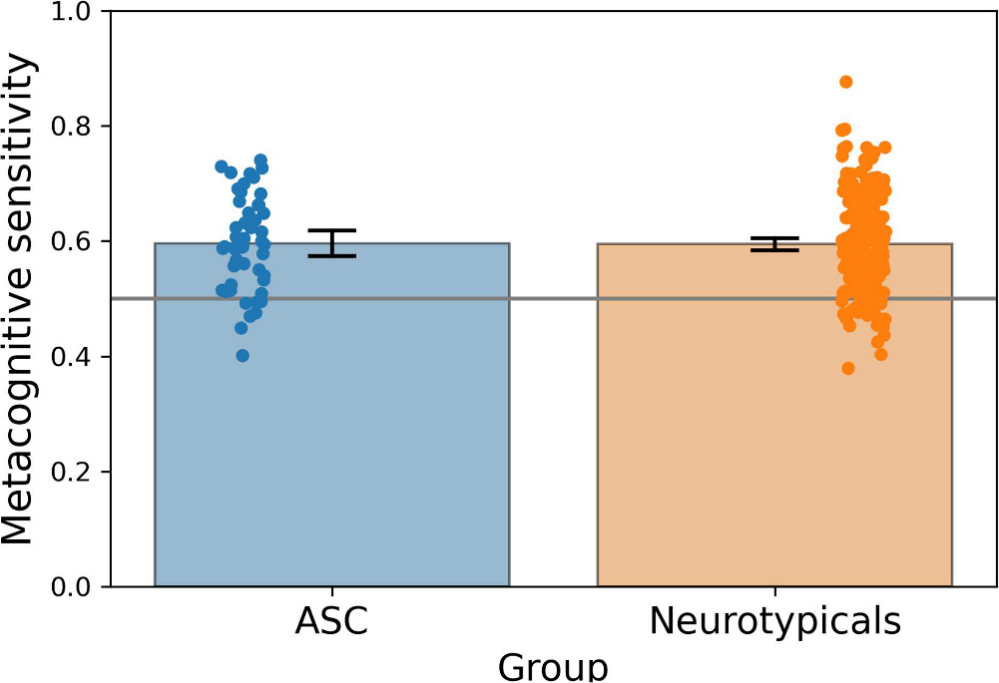
Mean type 2 ROC, metacognitive sensitivity in the odd-one-out-task across the ASC and neurotypical groups. The *bars* represent the 95% confidence intervals around the mean

Then assessed whether the ASC and the neurotypicals groups differed in their metacognition ability, namely, to evaluate the correctness of their responses in the odd-one-out task. Metacognitive ability was measured by a type 2 ROC analysis of how well confidence ratings discriminate between correct and incorrect responses (Fleming & Lau, 2014). The results showed no differences in metacognitive sensitivity across the two groups (*t*(276) = 0.072, $p = 0.943$; see Fig. 8).

### Assessing social preference biases in ASC

**Fig. 9:**
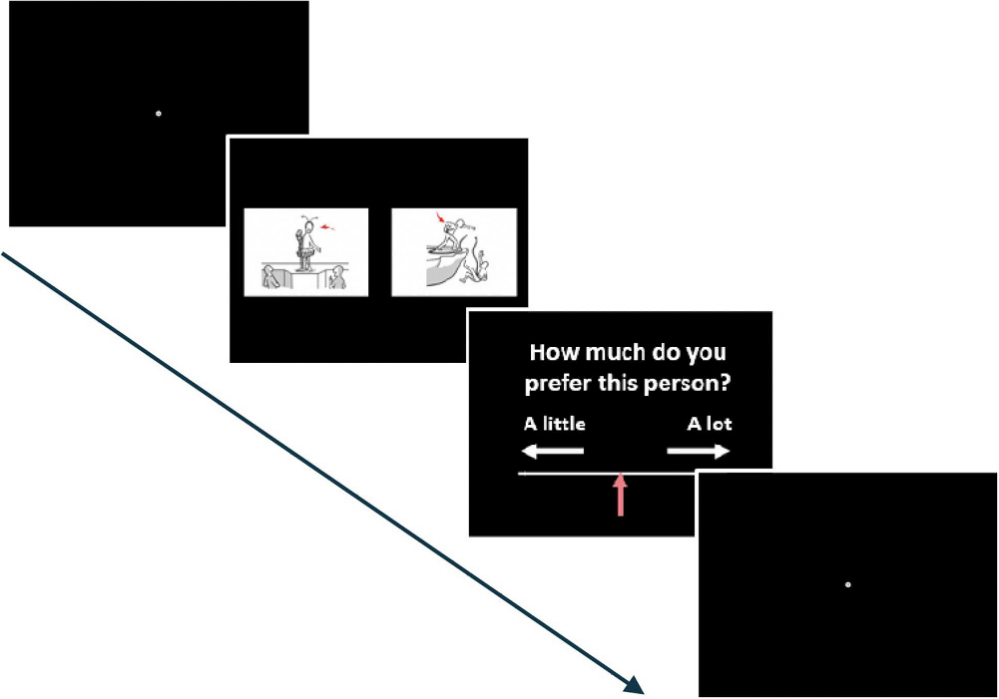
Example trial of the preference task. Two images were simultaneously presented, one on the left and one on the right side of the screen. One image depicted a socially desirable concept and the other a socially undesirable concept. Participants had to indicate which image protagonist they subjectively preferred. Following this, the adult participants rated their preference using a ten-point scale. This preference rating was not included in the children sample

Finally, we addressed biases in social preference by presenting participants with two images of opposite social desirability (i.e., desirable vs. undesirable), with the task being to indicate which image they subjectively preferred. The preference task is depicted in Fig. 9. Following the initial preference choice, participants made a second, more fine-grained rating using a 0 to 10-point scale.

**Fig. 10:**
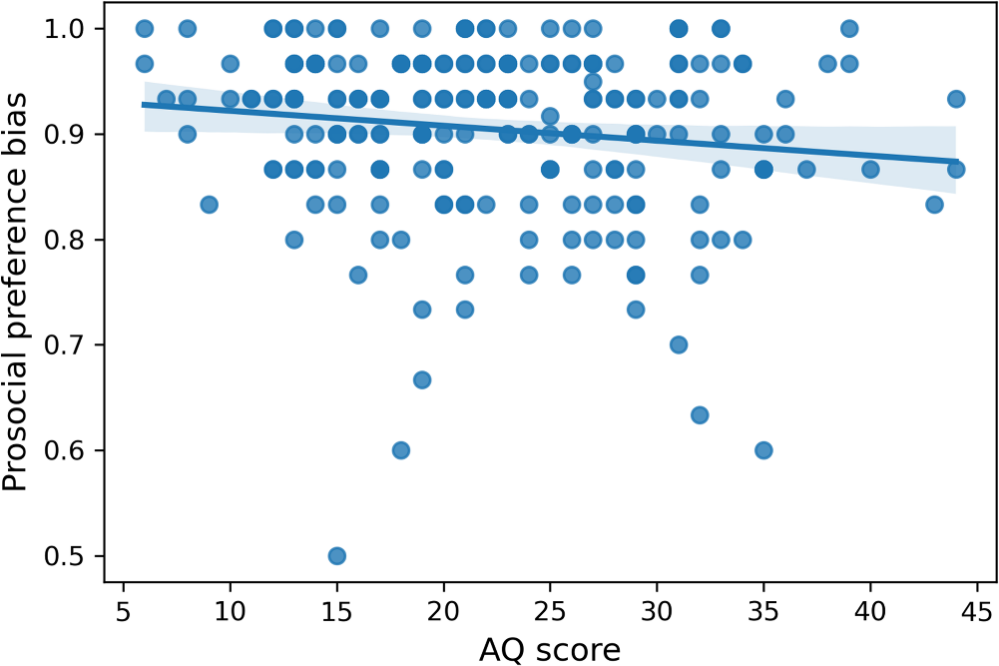
Scatter plot demonstrating a negative correlation between the AQ score and the probability of choosing a socially desirable image. The *bars* represent the 95% confidence intervals around the mean

We computed the mean probability of choosing the prosocial concept and correlated this probability with the individual AQ score of the neurotypical subjects. In light of the results obtained during the validation of the database, namely, those showing that participants ratings discriminate very well between the normative social desirability of the images (see Fig. 4), we discarded as an outlier one of the neurotypical participants with a probability of 0.2 of selecting the prosocial target. However, including all the data did not alter the pattern of results. Spearman’s rho indicated a significant negative correlation between the AQ score and the probability of a preference for the protagonist of a socially desirable concept (Spearman *r* = -0.15, $p = 0.024$), showing that neurotypical participants with higher AQ-scores were more likely to select the item associated with lower social desirability (Fig. 10).

**Fig. 11:**
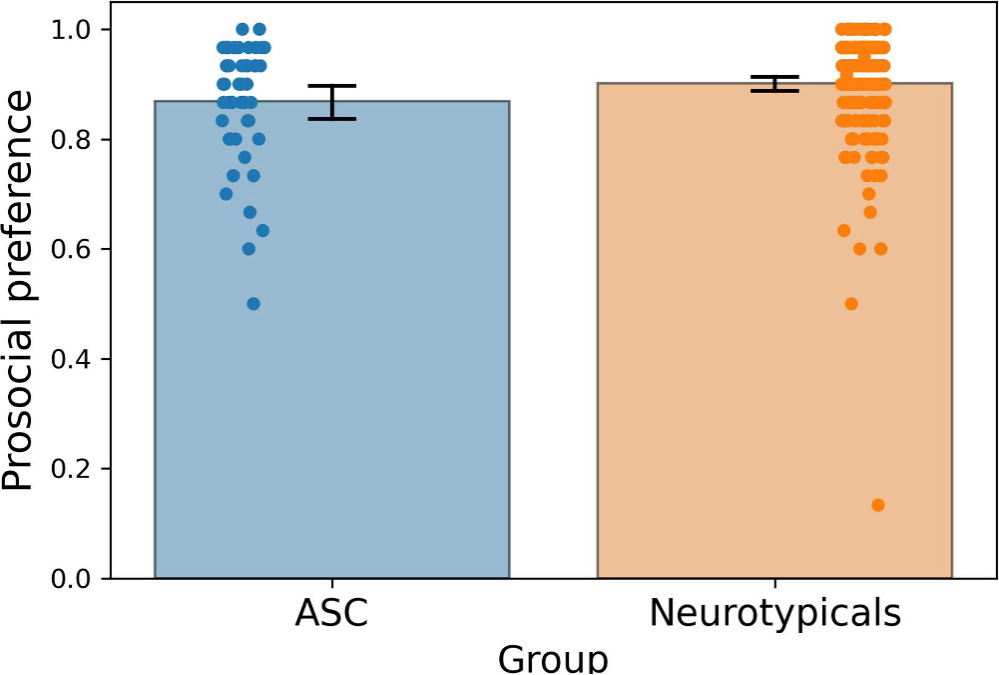
Differences in prosocial preference biases between the neurotypical and the ASC groups (adults). The *y*-axis represents the probability of choosing a socially desirable image. The *bars* represent the 95% confidence intervals around the mean

Then we compared preference biases between the neurotypical sample and the ASC sample. The likelihood that the ASC sample preferred the protagonist exhibiting a socially desirable behavior was lower compared to the neurotypical group (*t*(276) = 2.118, $p = 0.035$, Cohen’s *d* = 0.331; Mann–Whitney *U* = 4671.5, $p = 0.043$); we repeated the analysis following the removal of the neurotypical outlier with a preference of 0.2 (*t*(275) = 2.655, $p = 0.008$; Cohen’s *d* = 0.45; Mann–Whitney *U* = 4621.5, $p = 0.038$). These results are depicted in (Fig. 11).

Recall that after the primary preference choice, participants were asked to give a second rating on a ten-point scale (see Fig. 9). Overall, socially desirable choices were associated with significantly higher ratings (mean = 7.518; SD = 1.070) compared to the socially undesirable counterparts (mean = 5.301; SD = 2.136; *t*(249) = 18.207, $p < 0.001$; Wilcoxon signed-rank tests = 29922.5, $p < 0.001$). This means that the second-order rating somehow tracked the normative social desirability of the images. However, no differences between ASC and neurotypical groups were observed in how these second-order ratings related to the normative desirability of the selected concept (*t*(248) = 1.633, $p = 0.104$; Mann–Whitney *U* = 5414.5, $p = 0.209$).

**Fig. 12:**
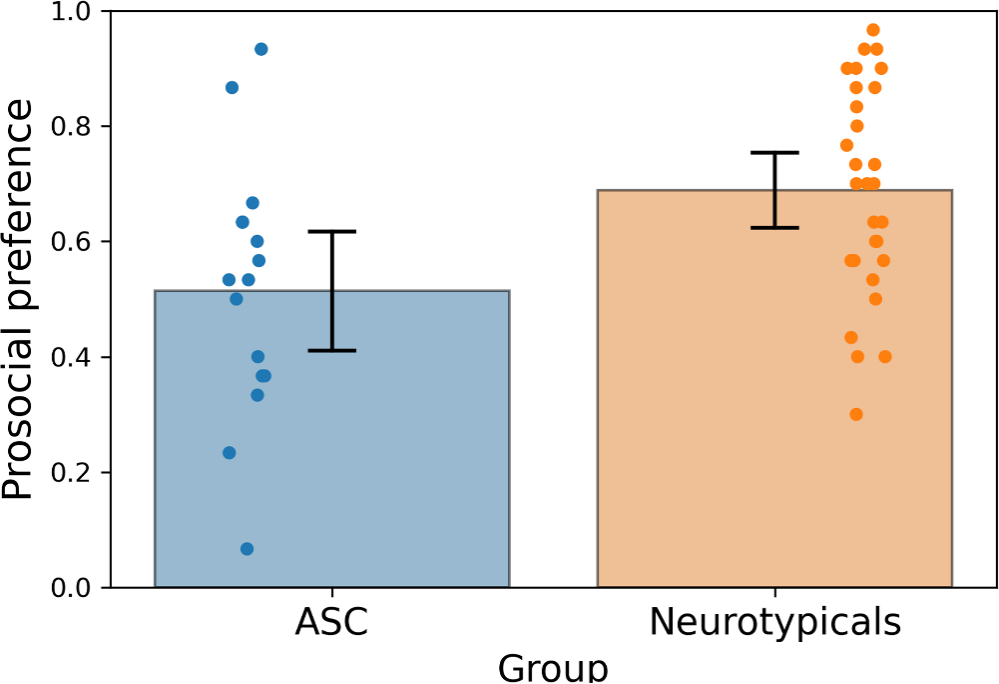
Differences in prosocial preference biases in children comparing the neurotypical and the ASC groups (adults). The *y*-axis represents the probability of choosing a prosocial image. The *bars* represent the 95% confidence intervals around the mean

Finally, we asked whether the preference bias is also present in children or whether the preference bias is more associated with adult samples and thereby more likely to reflect experience-dependent changes or developmental trajectories. We compared neurotypical children (*N* = 29, age range: 4–6) with a sample of ASC children (*N* = 16, age range: 4–9), matched as closely as possible in verbal mental age. In line with the patterns of results observed in adults, ASC children showed a significantly lower preference bias for socially desirable images compared to the neurotypicals (*t*(43) = 2.827, $p = 0.007$; Cohen’s *d* = 0.88; Mann–Whitney *U* = 124, $p = 0.011$). These results are depicted in Fig. 12.

We also re-analyzed the data considering the scores on each of the subscales of the ASQ (namely, social skill, attention switching, attention to detail, communication and imagination), but did not observe any reliable correlation between the subscale scores and the performance in the odd-one-out or the preference tasks (lowest *p* value of 0.143 for Spearman correlation of –0.097 between the preference bias and the imagination score.

### Gender effects

Although our study was not specifically designed to examine gender-related issues in our ASC sample (33 females, 16 males; gender data from one participant was missing), we performed some exploratory analyses in this regard considering that recent research points to the existence of a female ASC phenotype (Baldwin & Costley, 2016; Bargiela et al., 2016). Overall, the results showed that performance in the odd-one-out task and the pro-social preference bias were lower in males compared to females.^3^

## Discussion

We created and validated an image database of social concepts. The results of the semantic task demonstrated that the image database captures the meaning of abstract concepts and their normative social desirability (Anderson, 1968) consistently across participants. The standardization of the database can help to improve the consistency of experimental results, which may otherwise be hampered by different criteria for the selection and depiction of abstract concepts (Villani et al., 2019). This image database thus provides a standardized tool for investigating the representation of abstract social concepts in the fields of psycholinguistics, neuropsychology, and cognitive neuroscience. The image database can be used to develop novel experimental procedures that may or may not require conscious reasoning, for instance, tasks that merely rely on the automatic re-enactments of scripts based on depiction of situated behaviors and social experiences. Image databases like the present one are also required if the objective is to test children, populations with atypical linguistic development or neurological patients with language or semantic memory deficits, as they need less adjustments in cultural validations because they do not rely on written language. While some authors advocate for the role of language in the representation of abstract concepts in general (Borghi et al., 2018; Dove et al., 2020), they also hold that social experience is at least as important. The image database is composed of dispositional concepts (Ryle, 2009) that reflect how people would behave in certain situations, thereby promoting the re-enactment of the targeted concept and linked social experience (Matheson & Barsalou, 2018), which we believe is critical for investigating the representation of social concepts. A standardized image database to study abstract concepts has advantages over purely linguistic examples. For instance, it enables comparing knowledge representation across different neurodevelopmental and neuropsychological populations, as well as across different cultures and languages. One potential caveat is that introspective experiences and sensory-motor patterns associated with abstract conceptualizations may vary across cultures to some degree, and hence their universal representation may be challenging. Furthermore, we recognize the challenge of completely mitigating cultural biases related to the influence of actors’ gender in the images on participants’ judgments of social desirability. Despite our efforts to depict human figures that diverge from conventional Western representation norms, it is conceivable that connections with male actors may have arisen due to the prevalence of bald actors in the image database. Future studies using the database may assess the role of participant’s beliefs about the gender of the actors in the images regarding the conceptualizations of social desirability or affective representations of the images.

The experimental results showed that the representation of social concepts is dependent on individual neurodevelopmental traits linked to ASC. The results of the odd-one-out task, requiring the implicit identification of social desirability, showed no differences in performance between the ASC sample and the neurotypicals. ASC adults accurately extracted relevant social cues (bodily posture, mouth expressions, elements of situated experience in the image) to form an abstract conceptualization of social desirability.

However, the level of performance in the odd-one-out task was far from ceiling. It should be noted that participants did not receive explicit instructions regarding the nature of the odd-one-out item. Further, social desirability is one of many dimensions along which similarity can be computed across the randomly chosen triads, thereby making the odd-one-out task ambiguous. Furthermore, the ability to monitor the correctness of the responses in the odd-one-out task was similar across ASC and neurotypical samples. It has been debated whether ASC is associated with difficulties in metacognitive monitoring. Some studies indicated metacognitive deficits (Grainger et al., 2016b; Nicholson et al., 2021; van der Plas et al., 2021; Wilkinson et al., 2010), whereas other studies showed preserved metacognition in ASC (Grainger et al., 2016a; Maras et al., 2019; Sawyer et al., 2014). These prior studies were concerned with metacognition in perceptual, memory and reasoning tasks. The present results regarding metacognition for decisions involving social conceptualizations show no evidence for impaired metacognition in ASC.

Taken together, these observations are in keeping with prior studies showing that ASC individuals – including children – can exhibit skills relevant for social cognition such as mental state representation (e.g., intention recognition), or the ability to discriminate between social and moral transgressions (Carpenter et al., 2001; Grant et al., 2005; Shulman et al., 2012). However, Shulman and colleagues (Shulman et al., 2012) reported some differences in discriminatory ability between autistic and typically developing children. In particular, autistic children were more strict than typical children about conventional transgressions, which tended to be treated on a par with moral ones. Notwithstanding, the observation of preserved discrimination of the social desirability in ASC aligns with the view that some skills relevant for social cognition might be less impaired in ASC than previously thought (Chevallier et al., 2012).

Prior studies showed preference biases in ASC for nonsocial over social items (Crawford et al., 2016; Gale et al., 2019; Król & Król, 2020). Here, we examined preference biases in ASC within the social domain and showed that both adult and children samples of ASC participants had a stronger preference bias away from prosocial items towards competing socially undesirable items, compared to the neurotypical group (which also showed preference biases correlated with inter-individual variability in autistic trait). It is possible that the preference biases in ASC may be due to item-specific effects, for instance due to specific images that exemplify a non-prosocial behavior, but one that is not harmful or clearly anti-social in nature (e.g., not being part of some social situation). However, isolating item-specific effects in the present study is challenging because the combination of images in the preference and odd-one-out tasks was randomly generated on each trial, hence making it difficult to parse the contribution of specific images in the context of the other images presented.

The different pattern of responses that we observed in the ASC sample between the preference task and the odd-one-out task may shed some light on the factors underlying social difficulties in autism. Social cognition theories – such as accounts based on theory of mind deficits (Baron-Cohen et al., 1985) or Dawson’s early approach to autism (Dawson et al., 2004) – have long conceived of social difficulties as a consequence of difficulties in understanding other people’s mental lives, emotions, motivations, and so on. On these views, diminished social motivation would stem from underlying issues in understanding or manipulating social concepts. By contrast, more recent theories have argued that social difficulties relate primarily to diminished social motivation, which in turn reflects on fewer opportunities to interact with others and develop the relevant social cognition skills (Chevallier et al., 2012) – but see Jaswal and Akhtar (2019) for a recent critique. By administering the preference task and the odd-one-out task to the same groups of participants, we aimed to pry apart social cognition – i.e., “Which one of these images is socially (un)desirable?” – and social motivation – i.e., “Which one of these images do I subjectively prefer?”. Our results suggest that autistic participants may indeed exhibit diminished social motivation, given that they were less likely to select prosocial images with respect to their non-autistic counterparts. Yet, such a diminished motivation does not appear to affect social cognition, since the performance in the odd-one-out task did not differ between autistic and neurotypical participants. In this respect, social motivation theories are partially vindicated by our results, although our pattern of results also suggests that diminished social motivation does not necessarily imply diminished social cognition.

In any case, the observed pattern of results thus reveals similarities in social preference between different autistic populations: adults with typical verbal and cognitive skills, and children exhibiting a variety of verbal and cognitive skills. We propose that the lack of perceived reward in social interaction in ASC (Chevallier et al., 2012) explains the reduction in prosocial preference choices that we observed, which may in turn contribute to decreased motivation for social exposure in ASC (Chevallier et al., 2012). These results point to the importance of addressing the role of subjective experience in ASC beyond the standard neuropsychiatric symptoms and hypothesized biological markers (Liu & Lau, 2022). Due to how autistic individuals may experience the social world (i.e., unfamiliar, uncertain, or unsatisfying), they may be less sensitive to the social implications of behavior, and thus display choice biases on the basis of different considerations.

Exploratory analyses of gender in the ASC sample indicated that prosocial preference biases were higher in females. Although males tend to be diagnosed with ASC more often than females, recent work has drawn attention to the possible existence of a female autistic phenotype (Baldwin & Costley, 2016; Bargiela et al., 2016). One of the characteristic features of the female ASC profile would be higher social motivation and greater ability to form social relationships (Hiller et al., 2014; Sedgewick et al., 2019), which could potentially account for the higher prosocial bias and the higher identification performance of social desirability in the odd-one-out task in females compared to males. Additional work is needed to make further determinations. Future studies using the image database can further address how abstract social conceptualizations differ across different neuropsychological and psychiatric conditions in which social and affective processing may be compromised.

## Supplementary Information

Below is the link to the electronic supplementary material.

Supplementary file 1 (pdf 6847 KB)

## Data Availability

The image database, the associated computer vision model representations and the experimental data are available at https://osf.io/6myaf/

## Footnotes

1 https://www.kaggle.com/

2 Can pretrained ImageNet models generalize to sketches?

3 We compared the identification performance in the odd-one-out task between females and males, and also in connection with the preference biases. The results showed that odd-one-out identification performance was lower in males (mean = 0.515; SD = 0.147) compared to females (mean = 0.612; SD = 0.15) (*t*(47) = 2.14, $p = 0.038$, Cohen’s *d* = 0.652; Mann–Whitney *U* = 318, $p = 0.046$). We also observed that prosocial preference biases were lower in males (mean = 0.825; SD = 0.113) compared to females (mean = 0.897; SD = 0.092) (*t*(47) = 2.377, $p = 0.022$; Cohen’s *d* = 0.724; Mann–Whitney *U* = 376, $p = 0.016$). Interestingly, this pattern of results was observed despite the AQ score being similar between males (mean = 35.875, SD = 8.237) and females (mean = 37.06, SD = 8.61) (*t*(46) = 0.457, $p = 0.65$; Mann–Whitney *U* = 292, $p = 0.436$). These analyses were not performed in the children sample since there were only three females in the ASC group.
