# Correction to: A novel image database for social concepts reveals preference biases in autistic spectrum in adults and children

**DOI:** 10.3758/s13423-024-02629-7

**Published:** 2025-01-06

**Authors:** David Soto, Amaia Salazar, Patxi Elosegi, Antje Walter, Ning Mei, Ekaine Rodriguez, Valentina Petrollini, Agustín Vicente

**Affiliations:** 1 Basque Center on Cognition, Brain and Language, San Sebastian, Spain; 2 Ikerbasque, Basque Foundation for Science, Bilbao, Spain; 3 Department of Artistic Education, Complutense University of Madrid, Madrid, Spain; 4 University of the Basque Country- UPV/EHU, Leioa, Spain; 5 Shenzhen University, Nanshan District, Shenzhen, Guangdong Province, China

**Correction to : Psychonomic Bulletin & Review (2024) 31:1690–1703**

10.3758/s13423-023-02443-7

This paper has been updated with open access.

