# Supplementary material for: A novel image database for social concepts reveals preference biases in autistic spectrum in adults and children
Source: Psychon Bull Rev. 2024 Jan 18;31(4):1690–703. doi: 10.3758/s13423-023-02443-7 (PMC11779780; doi:10.3758/s13423-023-02443-7)
Supplement: Supplementary file 1 — (pdf 6847 KB) [file 13423_2023_2443_MOESM1_ESM.pdf]

Supplementary Figures

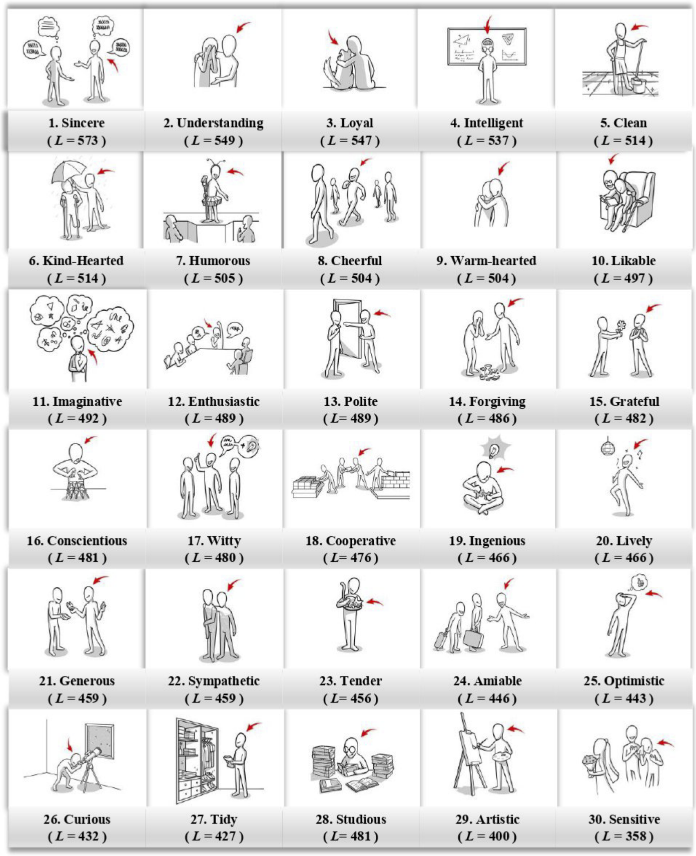

Supplementary Figure 1: *Image concepts associated with high social desirability.*

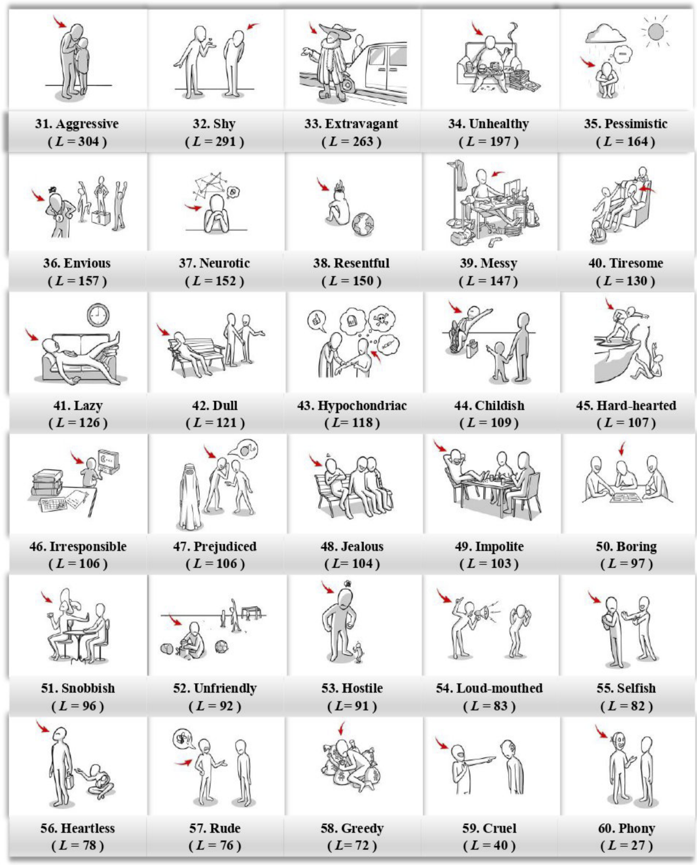

Supplementary Figure 2: *Image concepts associated with low social desirability.*

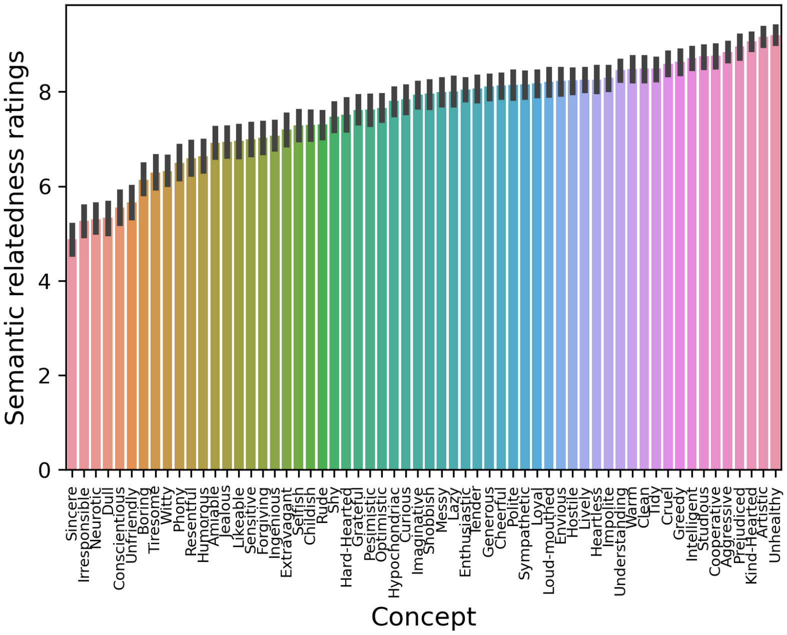

Supplementary Figure 3: *Average semantic relateness ratings across participants for each concept.*

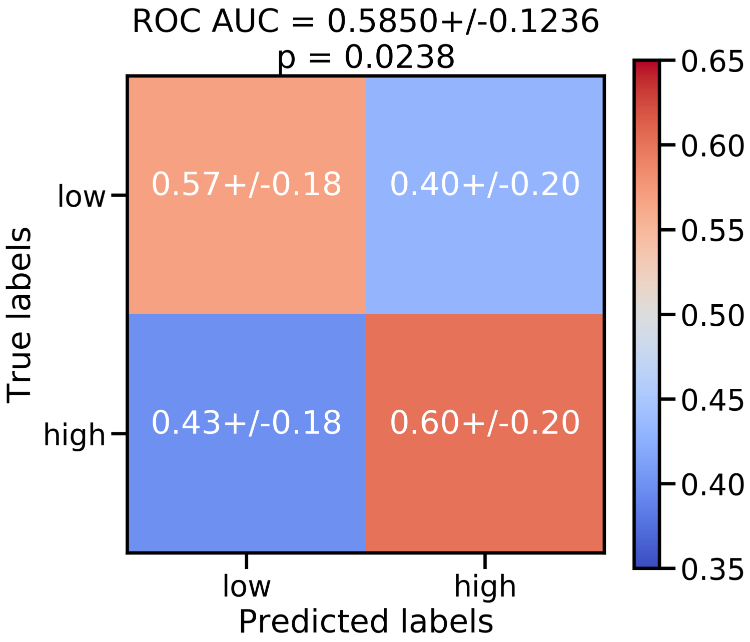

Supplementary Figure 4: *Decoding low vs. high social desirability from the hidden layer representations of the ResNext101 computer vision model.*

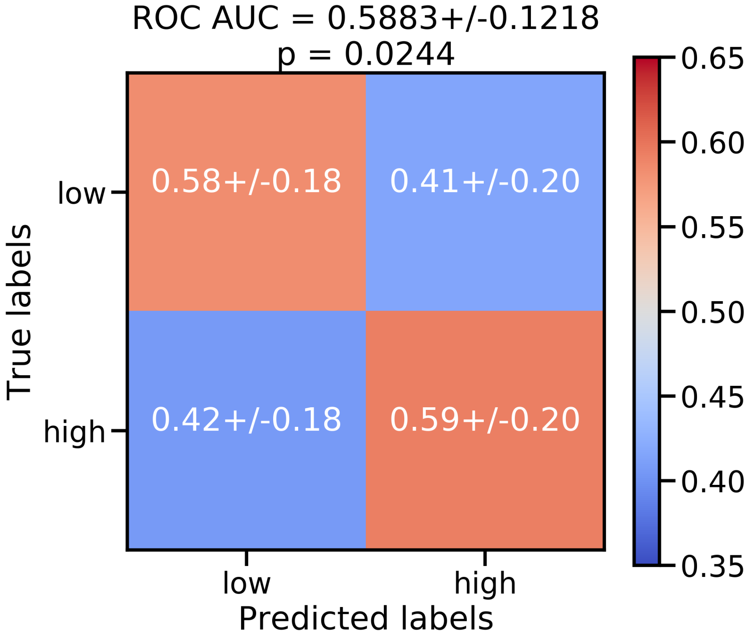

Supplementary Figure 5: *Decoding low vs. high social desirability from the first layer representations of the ResNext101 computer vision model.*

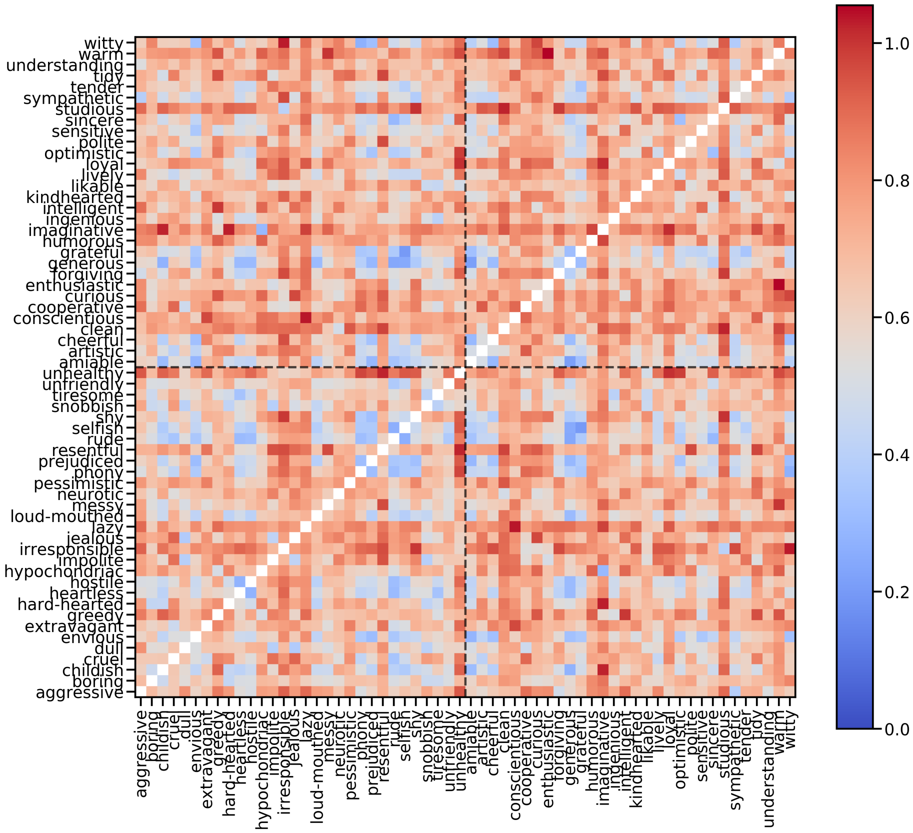

Supplementary Figure 6: *RDM of the hidden layer representations of the ResNext101 computer vision model.*

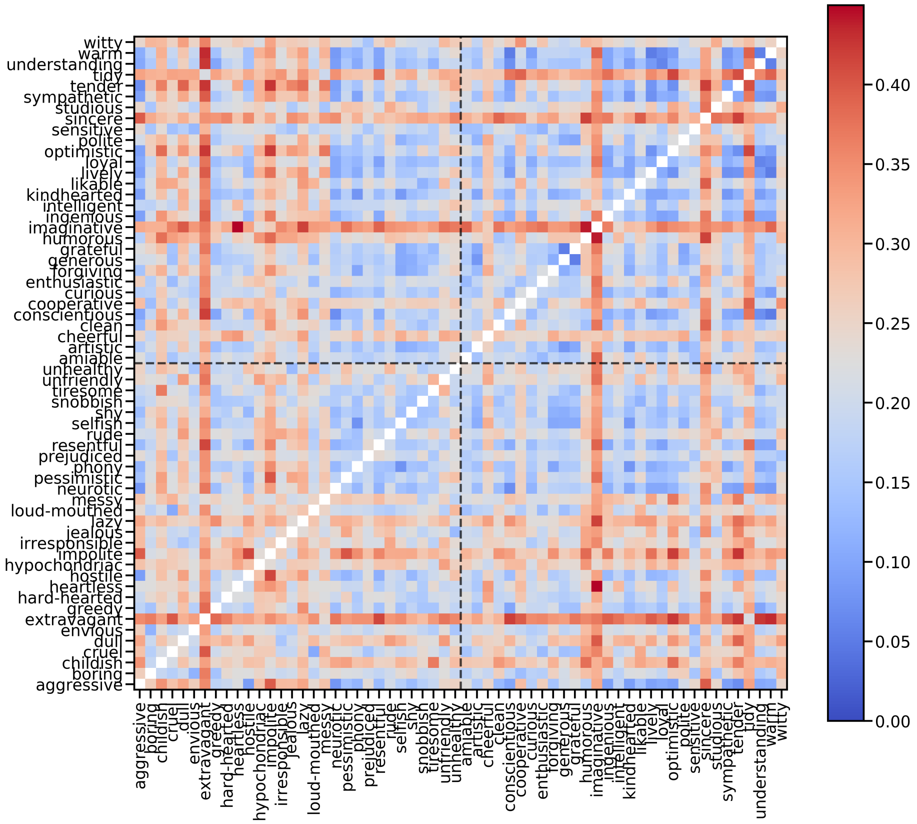

Supplementary Figure 7: *RDM of the first layer representations of the ResNext101 computer vision model.*
